# ASO Author Reflections: Extending Postoperative Observation of Oncogeriatric Surgery Patients After Hospital Discharge by Using Telemonitoring

**DOI:** 10.1245/s10434-021-09734-0

**Published:** 2021-02-27

**Authors:** Leonie T. Jonker, Barbara L. van Leeuwen

**Affiliations:** 1 Department of Surgical Oncology, University of Groningen, University Medical Centre Groningen, Groningen, The Netherlands; 2 Department of Epidemiology, University of Groningen, University Medical Centre Groningen, Groningen, The Netherlands

## Past

After oncogeriatric surgery, problems might occur in the vulnerable period after hospital discharge, where monitoring is less intensive than in the hospital.^1^ Furthermore, while the number of scientific publications on remote home monitoring has emerged rapidly in recent years, research on telemonitoring in the postoperative setting is relatively scarce.^2^ The few studies that investigated the potential of telemonitoring in this population have demonstrated its feasibility and an increase in patient engagement.^3^,^4^ To be better informed about (deviations in) recovery after surgery once patients are at home, more telemonitoring data should be collected.

## Present

In this study,^5^ we monitored physical activity, vital signs, and patient-reported symptoms in oncogeriatric surgical patients for 2 weeks after hospital discharge. In total, 24 (43%) patients experienced a complication at home, 13 of whom were readmitted to the hospital. This confirms the extent of the clinical problem we have addressed. Monitored parameters violated the threshold 329 of 5379 times (7%), most often because of physical inactivity. Patients with readmissions had more physical activity threshold violations. Unfortunately, no differences in threshold violations of vital signs and symptoms were observed between patients with and without postdischarge complications. Median pain score over 2 weeks was higher in patients with complications compared with patients without complications.

## Future

This study underlines the complexity of detecting complications after discharge in the oncogeriatric population. It requires more than the measurement of a single vital parameter, and it remains to be investigated how a combination of parameters affects the detection of complications and readmission rates. However, objectively measured physical activity might be of predictive value for postdischarge adverse events and should be explored in future studies. Finally, it is important to mention that telemonitoring in daily practice should not be considered a separate tool but rather a supplement to existing perioperative care.

## References

[1] Ommundsen N, Nesbakken A, Wyller TB (2018). Post-discharge complications in frail older patients after surgery for colorectal cancer. Eur J Surg Oncol.

[2] Farias FAC, Dagostini CM, Bicca YA (2020). Remote patient monitoring: a systematic review. Telemed J E Health.

[3] Metcalf M, Glazyrine V, Glavin K (2019). The feasibility of a health care application in the treatment of patients undergoing radical cystectomy. J Urol.

[4] Jonker LT, Plas M, de Bock GH (2021). Remote home monitoring of older surgical cancer patients: perspective on study implementation and feasibility. Ann Surg Oncol.

[5] Jonker LT, Lahr MMH, Oonk MHM, et al. Post-discharge telemonitoring of physical activity, vital signs, and patient-reported symptoms in older patients undergoing cancer surgery. *Ann Surg Oncol.* 2021. 10.1245/s10434-021-09707-3. 10.1245/s10434-021-09707-3 PMC7914037 33641013

